# High Quality of Infant Chondrocytes in Comparison with Adult Chondrocytes for Cartilage Tissue Engineering

**Published:** 2017-05

**Authors:** Fatemeh Mortazavi, Hajar Shafaei, Jafar Soleimani Rad, Leila Rushangar, Azadeh Montaceri, Masoud Jamshidi

**Affiliations:** 1 Department of Anatomy, Faculty of Medicine, Tabriz University of Medical Sciences, Tabriz, Iran;; 2 Pediatric Surgery Ward, Tabriz Children’s Hospital, Tabriz University of Medical Sciences, Tabriz, Iran

**Keywords:** Cartilage, Cell sheet, Tissue engineering, Infant, Chondrocytes, Adult

## Abstract

**BACKGROUND:**

Tissue engineering is used for the treatment of many diseases, and the ideal cell source for cartilage tissue engineering is chondrocytes. The main limitation of chondrocyte is the low number of cells in cartilage tissue engineering. This study investigated a suitable cell source with high proliferation rate to obtain a large number of chondrocytes.

**METHODS:**

Adult cartilage tissue samples were obtained from adult patients undergoing surgical procedure, and infant cartilage tissue samples were obtained from polydactyly surgical waste. After isolation and expansion of chondrocytes, the proliferation rate was evaluated by calculating population doubling time (PDT) and MTT assay for both types of cells. Cartilage film was prepared with sheets of over confluent chondrocytes. The cartilage tissue film from infant and adult chondrocytes were evaluated histologically and by immunefluorescent staining collagen type 2.

**RESULTS:**

PDT and MTT assays revealed that the growth rate of the infant chondrocytes was significantly higher than adult chondrocytes. Histological findings showed that sheets were thicker in the cartilage film of infant chondrocytes and they had more extracellular matrix between the sheets of cells than the cartilage film of adult chondrocytes. The findings of the immunofluorescent staining of cartilage film indicated that collagen type II film of polyductily was more positive than adult chondrocytes.

**CONCLUSION:**

The recent study presented a new cell source to overcome the limitation of low number of chondrocytes for cell therapy of cartilage defects in adults and also sheets of cells able to overcome the problems of scaffolds.

## INTRODUCTION

Osteoarthritis (OA) is the fourth important cause of inability and the current clinical cure strategies for articular cartilage include drug therapy, physiotherapy, surgery, autologous chondrocytes transplantation (ACT), mosaicplasty, and microfracture. Although these techniques effectively improve disease symptoms and relieve pain, but average long-term results are disappointing and do not improve joint function.^1^^-^^3^ ACT has been used to repair articular cartilage defects.^4^ Limitations of ACT include the low number of harvested cells, donor site morbidity, difficult surgical procedures, risks of pollution, and graft rejection.^5^

Tissue engineering is a new strategy that consists of the use of cells, scaffolds, and growth factors.^1^^,^^6^ The most common cell sources for cartilage tissue engineering are chondrocytes and mesenchymal stem cells (MSCs).^7^^,^^8^ There are challenges in application of MSCs such as differentiation variability.^1^^,^^9^ Therefore, chondrocytes are logical choices because they are found in native cartilage and play an important role in synthesizing, maintaining, and remodeling the extracellular matrix (ECM) of cartilage.^8^ Several studies have shown that 3D cultures of chondrocytes on scaffolds may help to maintain specific phenotype of chondrocytes.^10^^,^^11^ There are problems with biocompatibility of scaffolds.^12^ Cells sheet methods were developed in the 1990s that were able to overcome the problems of the scaffold defects.^13^^,^^14^ It has been shown that over confluent chondrocytes attach to other cells by extracellular proteins and act as a natural scaffold.^15^

According to previous studies, the proliferation rate of chondrocytes in young patients is higher than old ones.^16^ Surgical waste of polydactyl cartilage in children is a rich source of human chondrocyte. Therefore, in this study we aimed to compare the proliferation rate, morphology, and histologic structure of cartilage from infant and adult chondrocytes. The positive results of the current study will present new cell source that will remove limitations due to the number of cells for cell therapy in adult people. Since chondroblast obtained from surgical waste of polydactyl cartilage in children, to be used for transplantation in older people as allogeneic; Williams *et al.* during the four years study of human allograft transplant did not find any signs of immunological rejection.^17^

Another study reported survival rate of allograft patients after 5, 10 and 15 years, respectively to be 95%, 80% and 65%.^18^ Reasons for no rejection may be related to lack of blood vessels in the tissues that immune system does not have access to transplanted tissue.^19^ Another advantage of allograft transplant is that in the event of damage, allogeneic chondrocytes prepared, there will be links to the patient. While autologous transplantation will be required to have a surgery to remove cartilage samples and the next surgery will be needed to repair cartilage damage. We set to use film of cell sheets to overcome the disadvantage arising from the scaffold for cartilage tissue engineering as well.

## MATERIALS AND METHODS

Three samples with weight of 1 gr were obtained from the non-weight bearing articular cartilage of the adult knee joint (age: 52.3±9.7 years) undergoing arthroscopic procedure and polydactyly children (age: 25±16 months) with informed consent and ethical approval committee of the Tabriz University of Medical Sciences, Tabriz, Iran (ID: 5/4/1774). Cartilage samples were cut into 1–2 mm thick. For enzymatic digestion, cartilage pieces were transferred to conical tubes containing 2.5% pronase (Sigma, USA) and then shaken in water bath at 37°C for 1 hour. After incubation with pronase, enzymatic digestion was followed by 0.125% collagenase-2 (Gibco, USA) incubation in a shaking water bath at 37°C for 6-10 hours. 

After digestion, for neutralizing the collagenase enzyme, the same volume of working culture medium (DMEM/Gibco, USA) containing 10% FBS (Sigma, USA), 1% penicillin/streptomycin (Gibco, USA), and ascorbic acid (0.05 mg/ml) were added to the cell suspension. The digested samples were centrifuged at 1600 rpm/5 min, and then cell pellet was obtained. Before plating cells in culture flasks, the cells were counted under an invert microscope: Cells/ml=(Number of viable cells)/(Number of squares counted×dilution factor×10^4^); Total cell yield=(Cells/ml)×(Total volume of cell suspension). 

For expansion, the cells were plated at 7-10×0^4^ cells per T25 flask and kept at 37°C and 5% CO2. The first medium change was performed after 48 hours, and the following medium changes were three times per week. After the cells reached 80% confluence, the cells were subcultured. For passaging of cells, trypsin/EDTA (Sigma, USA) solution was used. Culture medium was taken away, and the cells were washed twice with sterile PBS (Sigma, USA). Then, 1 ml of trypsin/EDTA solution was added to each 25 ml culture flask, and then flasks were incubated at 37°C for 3 minutes. Two flasks were added 1 ml of working DMEM for neutralizing of trypsin/EDTA. Then, the cells were washed at 1600 rpm/5 min and counted for infant and adult chondrocytes. Population doubling time (PDT) was calculated by the following formula: 

$T_{d}=T\times \frac{\log2}{\log(\frac{N_{t}}{N_{o}})}$

Where N_0_ is the number of seeded cells at time t_0_ and N_t_ the number of taken cells at time t, and T represents the culture period_. _Infant and adult chondrocytes were cultured in plates with 96 wells and in 3000 cells per well. After 24 hours, 20 μl of MTT solution (5 mg/ml, Sigma USA) was added onto the chondrocytes. The cells were incubated at 37°C for 4 hours resulting in the formation of formazan crystal due to the action of succinate tetrazolium reductase belonging to mitochondrial respiratory chain on MTT. Then, 100μl of dimethyl sulfoxide (DMSO, Sigma, Germany) was added onto the crystals. The color absorbance was recorded by ELISA reader at 540 nm in a dark room. The standard curve was plotted for the known number of chondrocyte cells in infant and adult individuals.

**Fig. 1:**
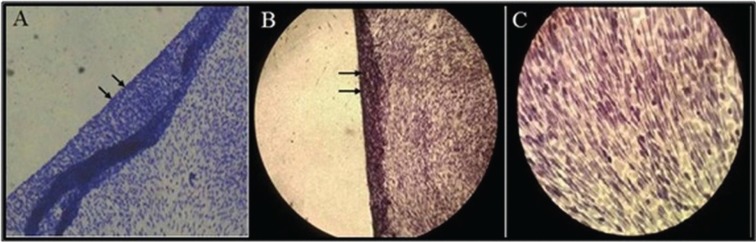
Removing the cell sheet formed on each cover slip after formation of extracellular matrix by chondrocytes, by PBS flashing. A: Toluidine blue staining; magnification, ×10, B: Hematoxylin and Eosin (H & E) staining; magnification, ×10, C: ×40

Chondrocytes were cultured on cover slips to prepare sheets of cell. Medium change was performed three times a week. After the formation of extracellular matrix by chondrocytes, sheets of cells were removed and placed in a tube (Figure 1). The culture medium was gently added onto the sheets and cultured for 2 weeks. After tissue formation, the constructs were prepared for histological evaluations. Formed tissues from infant and adult chondrocytes were fixed in 10% neutral buffered formalin for 1 hour and then embedded in paraffin. Sections of 5 μm thickness were prepared on the microtome (Leica, Germany). Newly formed ECM of tissue was evaluated through H&E staining.

After preparation of sections by the frozen section, tissue sections (3-5 µm-thick) were fixed by acetone for 5 min. Nonspecific antigens of samples were blocked using 10% mouse serum for 30 min at room temperature and then incubated with 1:100 of anti-collagen II antibody (ab3092, US) overnight in a humidified environment at 4°C to detect the existence of collagen types II. The secondary antibody was used (ab6563, US, 1:100) for 1 h in a humidified environment at 37°C. Subsequently nuclei were stained by DAPI and tissues were studied with florescent microscope. Positive and negative controls were included in the immunofluorescent staining protocol**.** All of Samples were studied with inverted microscope. Statistical analyses were performed using Graph Pad Prism. Mean comparison was carried out via one-way analysis of variance (*p*<0.05) was considered significant.

## RESULTS

**Fig. 2:**
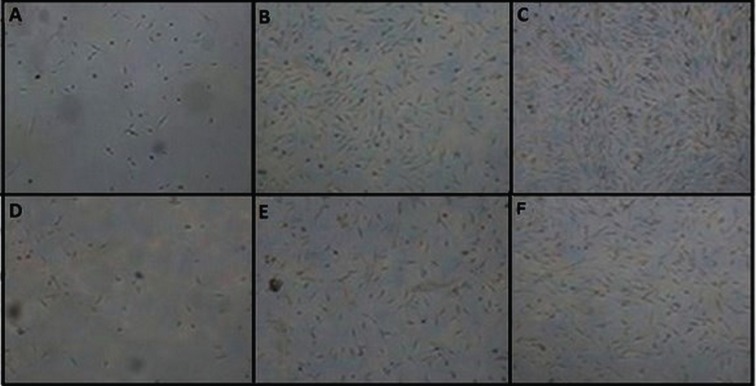
Morphology of chondrocytes at day 1, 3, and 5 (magnification, ×10). Infant chondrocytes at A: day 1, B: day 3, C: day 5. Infant chondrocytes reached confluence at day 5; adult chondrocytes at D: day 1, E: day 3, F: day 5. Infant chondrocytes reached confluence at day 10

**Fig. 3:**
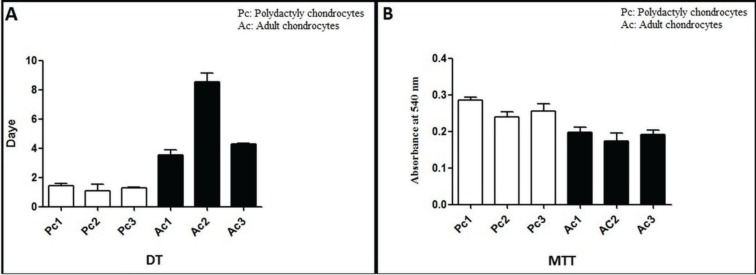
A: Population doubling time of chondrocytes in infant samples were compared with that of chondrocytes in adult samples. The growth rate of the infant chondrocytes was significantly higher than adult chondrocytes (*p*<0.05). B: MTT assays in infant and adult chondrocytes. MTT assays showed more mitotic cells in infant chondrocytes rather than adults (*p*<0.05

The confluence was reached in the infant chondrocyte flasks on the fifth day, and in the adult chondrocyte flasks, this was on day 10 for same number of seeded cells (Figure 2). The analysis of PDT data showed that variation in growth rate between adult chondrocyte donors was more than the variation between infant donors. Moreover, the growth rate of the infant chondrocytes was significantly higher than the adult chondrocytes (*p*<0.05). Also MTT assays showed more mitotic cells in infant chondrocytes than in adult chondrocytes and even this differences were statistically significant (*p*<0.05) between donors (Figure 3).

**Fig. 4:**
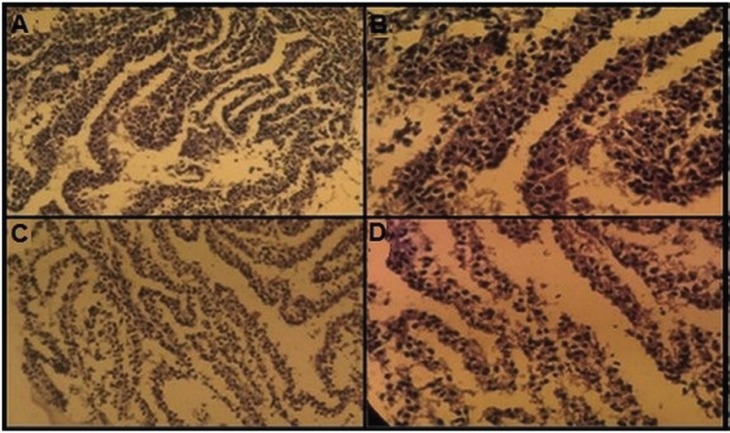
The film cartilage formation from infant and adult cells was studied histologically. The film cartilage of infant cells, magnification: A: ×10; B: ×40; The film cartilage of adult cells, magnification: C: ×10; D: ×40

**Fig. 5:**
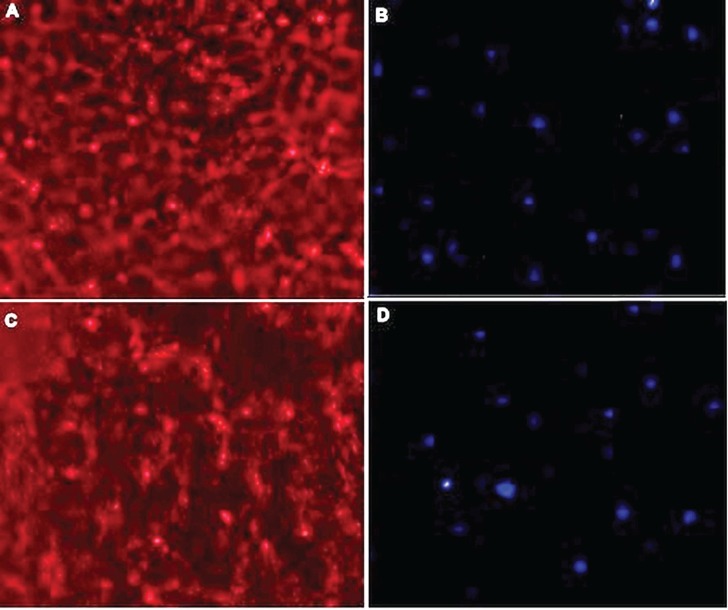
Immunofluorescent staining of the film cartilage formation from infant and adult cells. A: expression of collagen type II in cartilage film of polyductily; magnification ×40. B: Dapi, ×40, C: Expression

The findings of constructed tissue form infant and adult chondrocytes showed the cartilage film formation from the infant cell was larger than tissues produced by adult chondrocytes. Infant and adult chondrocyte sheets were studied histologically. Histologic H&E staining findings indicated that sheets were thicker in the cartilage film of infant chondrocytes and the cartilage film of infant chondrocytes had higher extracellular matrix than the cartilage film of adult chondrocytes between the cell sheets (Figure 4). The findings of the immunofluorescent staining indicated that collagen type II in cartilage film of polyductyly was more positive than adult (Figure 5).

## DISCUSSION

The source of cells for articular cartilage regeneration has been studied for several years.^16^ Common limitation of cartilage cell therapy is the low number of harvested cells during chondrocyte isolation.^10^ Finding the suitable cell source is the major challenge in tissue engineering.^8^ In this study, we concentrated on growth of the harvested chondrocyte cells from polydactyl infants, using 3D structure of newly formed extracellular matrix of chondrocytes as scaffold. To introduce the efficiency of these immature chondrocytes in film configuration, we compared their cell growth characteristics and tissue differences with adult chondrocytes. 

The findings showed that the growth rate of the cells from infants with polydactily was significantly higher than adult articular cartilage chondrocytes. Therefore the final results showed infant chondrocytes more suitable than adult chondrocytes. A pervious study has shown similar results;^16^ however, the current study worked on polyductyl chondrocyte for the first time, which is in agreement with the high activity of telomerase enzyme in prepubertal chondrocytes.^20^ Hence, a new cell source was presented that overcomes the problem of low number of chondrocytes for cell therapy. According to Rajagapal *et al.*, the iliac apophyseal growth plate in children is a good source of autolugus chondrocyte.^16^

Also, Maor *et al.* showed that chondrocytes derived from embryo mandibular cartilage have the ability to reproduce higher number of chondrocytes than adult tissues.^21^ Another limitation of chondrocyte is dedifferentiation of chondrocytes in monolayer expansion.^22^ Several research studies have shown that newly formed 3D cultures of chondrocytes in a scaffold may help the cells maintain specific phenotype.^10^^,^^11^ Unfortunately, there are some problems with regard to the biocompatibility of scaffolds preventing the clinical application of cartilage tissue engineering.^12^ The recent cell sheet methods were developed to overcome the problems of scaffold defects,^15^ using harvested cell sheets for various tissue reconstructions, including periodontal ligaments,^23^ cardiac patches,^24^ and bladder augmentation.^25^

Thus, they are potentially able to overcome the problems concerning the scaffold imperfections.^2^^,^^3^ This new technique introduced a new strategy for cartilage regeneration without a scaffold. In this study; the designed cartilage film in 10 sheets on both groups was studied histologically, indicating that the film cartilage formation from infant chondrocytes was larger than the sample predicted from the adults’ sample. Histological findings following H&E staining showed that the cartilage film produced by infants cells have higher ECM between the sheets of cells than film cartilage of adults. 

Comparison between cartilage film of polyductily and adult showed that expression of collagen type II in cartilage film of polyductily was more positive than adult. The final results showed that the quality of infant chondrocytes was better than adult chondrocytes for cartilage tissue engineering. The present study introduced a new cell source that overcomes the limitations of low number of cells for cell therapy in adult individuals and also the design of cartilage film to form sheets of cells able to overcome the problems of scaffolds. However, in vivo evaluations, it is needed to show heterologous transplantation outcomes.
